# Prevalence and Correlates of (Internet) Gaming Disorder among Young Adults in Singapore

**DOI:** 10.1007/s11126-025-10119-9

**Published:** 2025-02-12

**Authors:** Peter K. H. Chew, Kuhanesan N. C. Naidu, Jing Shi, Melvyn W. B. Zhang

**Affiliations:** 1 James Cook University, Singapore, Singapore; 2 Department of Psychological Medicine, National University Health System, Singapore, Singapore; 3 Saw Swee Hock School of Public Health, National University of Singapore, Singapore, Singapore; 4 Department of Health and Social Science, Singapore Institute of Technology, Singapore, Singapore; 5 Nanyang Technological University, Singapore, Singapore

**Keywords:** Prevalence, Risk Factors, Behavioral Addiction, Internet Gaming Disorder, DSM-V, ICD-11

## Abstract

There is a lack of estimates of prevalence rates of Internet gaming disorder (IGD) and gaming disorder (GD) among young adults in Singapore. Consequently, the current study aimed to examine the prevalence rates of IGD and GD, and their relationships with known correlates. Participants were a representative sample of 1008 young adults in Singapore. They completed instruments that assess IGD, GD, gaming motivations, negative emotional states, and sleep quality. The results showed a prevalence rate of 10.3% for IGD and 5.0% for GD. Males had a higher IGD and GD prevalence rate (14.6% and 7.2%) than females (6.2% and 2.8%). Furthermore, participants with IGD or GD reported higher gaming time, and higher scores on gaming motivations, negative emotional states, and sleep quality than their counterparts with no IGD or GD. The higher prevalence rates in this study compared to other global studies suggest that problematic gaming is a social issue in Singapore. Known correlates relating to gender, gaming time, motivations, and emotions were confirmed in the current study. The finding that participants with IGD or GD had better sleep quality was inconsistent with previous studies and could be due to the use of a single item to assess sleep quality. Limitations include the use of a cross-sectional correlational design. Future research could develop and evaluate prevention programs or intervention programs to alleviate the symptoms and negative consequences of problematic gaming.

**Supplementary Information:**

The online version contains supplementary material available at 10.1007/s11126-025-10119-9.

## Prevalence and Correlates of (Internet) Gaming Disorder among Young Adults in Singapore

Being a small city-state and the smallest island country in Southeast Asia, Singapore has historically strongly valued academic and employment success, hard work, and strong work ethic to bring prosperity to the country and its citizens [19]. Mental health subjects such as behavioral addictions and problematic behaviors are taboo topics and are stigmatized in society [19]. In contrast, video games and mobile gaming are the fastest growing industries (and one of the most lucrative) in Southeast Asia with an increasing number of people reporting they are concerned about Internet addiction and detriments to physical health [40–47]. With Internet gaming disorder (IGD) being added to the *Diagnostic and Statistics Manual of Mental Disorders*, 5th edition (DSM-V) as a condition that warrants further study and gaming disorder (GD) being added to the *International Classifications of Diseases*, 11th edition (ICD-11), Singapore is behind in establishing prevalence rates and risk factors to determine the extent this possible public health concern (American Psychiatric Association [2]; World Health Organization [50])^1^.

IGD has been defined as “a pattern of excessive and prolonged Internet gaming that results in a cluster of cognitive and behavioral symptoms, including progressive loss of control over gaming, tolerance, and withdrawal symptoms, analogous to the symptoms of substance use disorders” (American Psychiatric Association [2], p. 796). Specifically the nine criteria are: (1) preoccupation with gaming, (2) withdrawal symptoms like irritability or anxiety when unable to play games, (3) tolerance – the need to increase time spent on games, (4) unsuccessful attempts to reduce or stop gaming, (5) loss of interest in other activities because of gaming, (6) continued gaming despite problems, (7) deceiving family members or others about amount of gaming, (8) gaming to escape or to relieve negative moods, and (9) risk or loss of a relationship, job, or educational or career opportunity because of gaming. Individuals who meet five or more criteria during the past 12 months would meet the diagnostic criteria for IGD.

GD has been defined as a pattern of persistent gaming behavior, either online or offline, that includes the following features: (1) impaired control over gaming, (2) increased priority given to gaming over other activities, (3) continued gaming despite problems, and (4) impairment in various life domains (e.g., family, educational, occupational, etc.) (World Health Organization [50]). Individuals who meet all of the criteria during the past 12 months would meet the diagnostic criteria for GD. Currently, because the ICD-11 GD criteria is relatively new and not used frequently in research, most of the studies that were included have been based on the DSM-V IGD criteria.

The common correlates relating to IGD include gender, gaming time, and gaming motivations. Overall, with some exceptions [3], males tend to be at higher risk for IGD than females [11, 31, 36]. Indeed, a meta-analysis of IGD and social media addiction research showed that males are at risk for GD whereas females are at risk for problematic social media use [42]. With regards to gaming time, a large-scale study consisting of 123,262 participants from 168 countries found a positive association between gaming time and the number of endorsed DSM-5 Internet gaming criteria [33]. Finally, although there are many conceptualizations of gaming motivations [10], the current study used the seven factors derived from the Motives for Online Gaming Questionnaire: (a) social, (b) escape, (c) competition, (d) coping, (f) skill development, (g) fantasy, and (h) recreation [13]. Studies have shown that all seven factors are positively correlated with gaming disorder [27, 35].

There are other negative correlates to IGD that have been studied. For example, IGD is positively correlated with negative emotional states and stress, and increases one’s predisposition towards depression and anxiety [44, 49]. In fact, individuals with IGD had higher depression, anxiety, and stress than their counterparts without IGD [4]. In addition, IGD is associated with poorer sleep quality where IGD was found to significantly predict poor sleep quality after controlling for demographic variables like age and gender [49]. A mediation study showed that IGD leads to poorer sleep quality via lower impulse control and higher bedtime procrastination [25]. Overall, IGD is associated with a wide range of negative consequences like financial, health, psychological, social, and work/study harm [8].

Given the wide range of prevalence rates for problematic gaming globally, meta-analyses have been conducted to statistically synthesize the data. A meta-analysis of 53 studies found prevalence rates that ranged from 0.16 to 21.76%, with a pooled prevalence rate of 3.05% [41]. Another meta-analysis of 71 studies found prevalence rates that ranged from 0.30 to 17.70%, with a pooled prevalence rate of 3.30% [22]. Both meta-analyses also found that participants from Asian regions had higher prevalence rates than participants from other regions like Europe (5.08% vs. 2.72%; [41] and North America (6.30% vs. 3.60%; [22]. With regards to gender, males had higher prevalence rates (6.31%) than females (2.54%) [41]. Finally, younger participants tend to be at risk for problematic gaming. Indeed, a meta-analysis of 155 studies involving adolescents and young adults only found pooled prevalence rates of 8.80% and 10.40%, respectively [17]. However, none of the studies in the meta-analyses used the relatively new ICD-11 GD criteria [50]. A recent study involving 560 participants from China and the UK used the ICD-11 GD criteria and found a prevalence rate of 1.80% only [33]. Clearly, more research is needed to understand the discrepancy in prevalence rates between the two sets of diagnostic criteria.

Few large-scale studies have been conducted to examine the prevalence rates of problematic gaming in Singapore. This is surprising given the high Internet penetration rate of 99% [20] and high percentage of gamers (up to 90% among 18 to 24 year olds) [51] in Singapore. One study involving 2998 primary and secondary school students found a prevalence rate of 8.70% [12]. Another study found a 15.40% prevalence rate among 1107 college students [45]. However, these two studies did not assess problematic gaming using established criteria by either the DSM-V or ICD-11, imposing a limit to the utility of their findings. There is one study that used the DSM-V IGD criteria and found a prevalence rate of 17.70% among 1251 adolescents and young adults in Singapore [43]. Nevertheless, despite its use of appropriate diagnostic criteria, there are three limitations associated with the study. First, the study recruited individuals who play online games only. Despite the term ‘Internet’ in Internet gaming, the DSM-V has made explicit that the condition could involve offline games too [2]. Consequently, the exclusion of those who play offline games only imposed a limit to the generalizability of their findings. Second, because the study preceded the development of the ICD-11 GD criteria [50], it was unable to provide an estimate of prevalence rates based on this new criteria. Finally, their data was collected in 2014 which is now outdated, given the speed at which games and technology develop. Given the increasing number of gamers and gaming revenue [21, 48] and the increased risk for problematic gaming during the COVID-19 pandemic [23, 24, 26] recent data is needed to provide a better understanding of the phenomenon.

The current study aimed to provide an estimate of the prevalence rates and the state of gaming in Singapore in 2023 using both DSM-V and ICD-11 criteria to address the limitations of prior studies. The study focused on young adults only (defined as between 18 and 40 years of age) [16, 30] since they are at higher risk for problematic gaming [17]. This study also aimed to examine the relationships between the instruments based on both of these criteria. Finally, known correlates (gender, gaming time, and gaming motivations) and negative correlates (negative emotional states and poor sleep quality) to IGD and GD will be examined.

## Method

### Participants

Participants were a representative sample of young adults recruited by a survey panel based on the inclusion and exclusion criteria. Specifically, participants should be (a) Singaporeans or permanent residents, (b) played at least one game in the past 12 months [33, 43] and (c) between 18 and 40 years of age. A total of 1560 participants were recruited. However, 73 (4.7%) did not provide informed consent, 20 (1.3%) did not complete the screener question, 200 (12.8%) did not play games in the past 12 months, 61 (3.9%) were not Singaporeans or permanent residents, 38 (2.4%) were not between 18 and 40 years of age, and 160 (10.3%) had missing data on one or more instruments. These cases were removed, resulting in a total of 1008 participants (49.8% females; 74.1% Chinese, 13.4% Malays, 9.3% Indians, and 3.2% Others). Their age ranged from 18 to 40 years (*M* = 28.46, *SD* = 6.20). The gender and ethnic distribution is similar to the Singaporean population (51.1% females; 74.3% Chinese, 13.5% Malays, 9.0% Indians, and 3.2% Others) [38]. The demographic and gaming-related information of the samples are presented in Tables 1 and 2, respectively.

**Table 1:** Demographic information of the Sample, n (%)

Variables	Total Sample	IGD Prevalence		GD Prevalence	
	*n* = 989 to 1008	Yes (*n* = 103 to 104)	No (*n* = 886 to 904)	Yes (*n* = 49 to 50)	No (*n* = 940 to 958)
Nationality					
Singaporean	885 (87.8)	91 (87.5)	794 (87.8)	47 (94.0)	838 (87.5)
Permanent Resident	123 (12.2)	13 (12.5)	110 (12.2)	3 (6.0)	120 (12.5)
Age, *M* (*SD*)	28.46 (6.20)	28.17 (5.22)	28.49 (6.31)	27.54 (6.14)	28.51 (6.20)
Gender					
Male	499 (49.5)	73 (70.2)	426 (47.1)	36 (72.0)	463 (48.3)
Female	502 (49.8)	31 (29.8)	471 (52.1)	14 (28.0)	488 (50.9)
Prefer not to say	7 (0.7)	0 (0.0)	7 (0.8)	0 (0.0)	7 (0.7)
Ethnicity					
Chinese	747 (74.1)	68 (65.4)	689 (75.1)	32 (64.0)	715 (74.6)
Malay	135 (13.4)	20 (19.2)	115 (12.7)	11 (22.0)	124 (12.9)
Indian	94 (9.3)	13 (12.5)	81 (9.0)	6 (12.0)	88 (9.2)
Others	32 (3.2)	3 (2.9)	29 (3.2)	1 (2.0)	31 (3.2)
Housing Type					
1-Room HDB Flat	13 (1.3)	6 (5.8)	7 (0.8)	4 (8.0)	9 (0.9)
2-Room HDB Flat	32 (3.2)	1 (1.0)	31 (3.4)	1 (2.0)	31 (3.2)
3-Room HDB Flat	275 (27.3)	24 (23.1)	251 (27.8)	14 (28.0)	261 (27.3)
4-Room HDB Flat	315 (31.3)	38 (36.5)	277 (30.7)	16 (32.0)	299 (31.2)
5-Room HDB Flat	180 (17.9)	14 (13.5)	166 (18.4)	6 (12.0)	174 (18.2)
Condominium	160 (15.9)	16 (15.4)	144 (15.9)	7 (14.0)	153 (16.0)
Landed Properties	28 (2.8)	4 (3.8)	24 (2.7)	2 (4.0)	26 (2.7)
Others	4 (0.4)	1 (1.0)	3 (0.3)	0 (0.0)	4 (0.4)
Occupation Status					
Student	179 (17.8)	10 (9.6)	169 (18.7)	6 (12.0)	173 (18.1)
Employed	770 (76.5)	90 (86.5)	680 (75.4)	44 (88.0)	726 (75.9)
Unemployed	36 (3.6)	4 (3.8)	32 (3.5)	0 (0.0)	36 (3.8)
Others	21 (2.1)	0 (0.0)	21 (2.3)	0 (0.0)	21 (2.2)
Education Level					
Below Secondary	2 (0.2)	0 (0.0)	2 (0.2)	0 (0.0)	2 (0.2)
Secondary	37 (3.7)	6 (5.8)	31 (3.5)	2 (4.1)	35 (3.7)
Post-Secondary (Non-Tertiary)	61 (6.2)	11 (10.7)	50 (5.6)	5 (10.2)	56 (6.0)
Diploma and Professional Qualification	211 (21.3)	23 (22.3)	188 (21.2)	12 (24.5)	199 (21.2)
University	678 (68.6)	63 (61.2)	615 (69.4)	30 (61.2)	648 (68.9)

Note. HDB = Housing and Development Board; The sample sizes varied due to missing data on some demographic variables

**Table 2:** Gaming-related information of the Sample, M (SD)

Variables	Total Sample	IGD Prevalence		GD Prevalence	
	*n* = 969 to 1008	Yes (*n* = 97 to 104)	No (*n* = 872 to 903)	Yes (*n* = 43 to 50)	No (*n* = 926 to 957)
Gaming Time					
Weekday	2.99 (2.75)	4.52 (3.91)	2.82 (2.53)	5.43 (4.70)	2.88 (2.57)
Weekend	4.31 (3.22)	6.46 (4.83)	4.07 (2.89)	6.70 (4.38)	4.19 (3.11)
Money Spent on Games	206.78 (536.12)	249.39 (463.12)	201.87 (543.91)	442.36 (555.51)	194.47 (532.52)
Favorite Game Genre, *n* (%)					
Action	206 (20.4)	30 (28.8)	176 (19.5)	13 (26.0)	193 (20.1)
Adventure	187 (18.6)	14 (13.5)	173 (19.1)	4 (8.0)	183 (19.1)
Board	40 (4.0)	1 (1.0)	39 (4.3)	1 (2.0)	39 (4.1)
Card	36 (3.6)	3 (2.9)	33 (3.7)	1 (2.0)	35 (3.7)
Casino	27 (2.7)	4 (3.8)	23 (2.5)	4 (8.0)	23 (2.4)
Casual	43 (4.3)	4 (3.8)	39 (4.3)	1 (2.0)	42 (4.4)
Educational	19 (1.9)	5 (4.8)	14 (1.5)	3 (6.0)	16 (1.7)
Music	81 (8.0)	8 (7.7)	73 (8.1)	7 (14.0)	74 (7.7)
Puzzle	60 (6.0)	2 (1.9)	58 (6.4)	3 (6.0)	57 (5.9)
Racing	22 (2.2)	2 (1.9)	20 (2.2)	2 (4.0)	20 (2.1)
Role Playing	84 (8.3)	8 (7.7)	76 (8.4)	5 (10.0)	79 (8.2)
Simulation	52 (5.2)	2 (1.9)	50 (5.5)	1 (2.0)	51 (5.3)
Sports	48 (4.8)	7 (6.7)	41 (4.5)	0 (0.0)	48 (5.0)
Strategy	68 (6.7)	11 (10.6)	57 (6.3)	4 (8.0)	64 (6.7)
Trivia	4 (0.4)	0 (0.0)	4 (0.4)	0 (0.0)	4 (0.4)
Word	12 (1.2)	1 (1.0)	11 (1.2)	0 (0.0)	12 (1.3)
Others	19 (1.9)	2 (1.9)	17 (1.9)	1 (2.0)	18 (1.9)

The sample sizes varied due to missing data on some gaming-related variables

## Instruments

### The Background Information Form

A background information form was developed to collect demographic and gaming-related information. Demographic variables included nationality, age, gender, ethnicity, housing type, occupation, and current/highest education level. Gaming-related variables included the average amount of time (in hours) spent playing games in a typical weekday and weekend, average amount of money (in SGD) spent on games in a month, and favorite game genre. For the latter, only game genres that were found in both the Apple and Google app store are listed for selection.

## The Internet Gaming Disorder Scale-Short-Form (IGDS9-SF)

The IGDS9-SF is a 9-item instrument designed to assess the nine criteria of IGD in the DSM-V: (a) preoccupation, (b) withdrawal, (c) tolerance, (d) unsuccessful attempts to stop, (e) loss of interest in other activities, (f) continued gaming despite problems, (g) deception, (h) relieve negative moods, and (i) loss of a relationship or job [32]. Participants are asked to report on their gaming activity during the past 12 months. Responses are made on a 5-point Likert scale that ranges from 1 = *Never* to 5 = *Very Often*. Responses given as 4 or 5 (*Often* or *Very Often*) are coded as an endorsement of the criterion. An endorsement of five or more criteria suggests the presence of IGD and is used as an indicator of IGD prevalence. The unidimensional structure of the instrument has been supported by exploratory and confirmatory factor analysis [32]. In addition, the instrument had an acceptable internal consistency of 0.87. In the current study, the instrument had an acceptable internal consistency of 0.93.

## The Gaming Disorder Test (GDT)

The GDT is a 4-item instrument designed to assess the four criteria of GD in the ICD-11: (a) impaired control over gaming, (b) increasing priority given to gaming, (c) continued gaming despite problems, and (d) impairment in various life domains [33]. Participants are asked to report on their gaming activity during the past 12 months. Responses are made on a 5-point Likert scale that ranges from 1 = *Never* to 5 = *Very Often*. Responses given as 4 or 5 (*Often* or *Very Often*) are coded as an endorsement of the criterion. An endorsement of all four criteria suggests the presence of GD and is used as an indicator of GD prevalence. The unidimensional structure of the instrument has been supported by confirmatory factor analysis [33]. In addition, the instrument had an acceptable internal consistency of 0.84. In the current study, the instrument had an acceptable internal consistency of 0.90.

### The Motives for Online Gaming Questionnaire

The Motives for Online Gaming Questionnaire is a 27-item instrument designed to assess seven factors of gaming motivations: (a) social, (b) escape, (c) competition, (d) coping, (f) skill development, (g) fantasy, and (h) recreation [13]. Responses are made on a 5-point Likert scale that ranges from 1 = *Almost Never/Never* to 5 = *Almost Always/Always*. Appropriate item scores are summed for each factor, with higher scores indicating higher levels of the respective gaming motivation. Scores for each factor range from 4 to 20 (except for recreation which range from 3 to 15). The seven-factor structure of the instrument has been supported by exploratory and confirmatory factor analysis [13]. In addition, the factors had acceptable internal consistencies that ranged from 0.79 to 0.90. In the current study, the factors had acceptable internal consistencies that ranged from 0.82 to 0.88.

## The Depression Anxiety Stress Scale

The Depression Anxiety Stress Scale is a 12-item instrument designed to assess three factors of negative emotional states: (a) depression, (b) anxiety, and (c) stress [1]. Responses are made on a 4-point Likert scale that ranges from 0 = *Did not apply to me at all* to 3 = *Applied to me very much*,* or most of the time*. Appropriate item scores are summed for each factor, with higher scores indicating higher levels of the respective negative emotional state. Scores for each factor range from 0 to 12. The three-factor structure of the instrument has been supported by confirmatory factor analysis [1]. In addition, the factors had acceptable internal consistencies that ranged from 0.66 to 0.85. In the current study, the factors had acceptable internal consistencies that ranged from 0.83 to 0.89.

## The Single-Item Sleep Quality Scale

The Single-Item Sleep Quality Scale is a visual analogue scale designed to assess sleep quality [39]. Responses are made on an 11-point scale with the following labels: 0 = *terrible*, 1 to 3 = *poor*, 4 to 6 = *fair*, 7 to 9 = *good*, and 10 = *excellent*, with higher scores indicating higher sleep quality. The scale is highly correlated with the Pittsburgh Sleep Quality Index (*r* = − .88 to − 0.92) [7] and is able to discriminate between those who sleep normally from those with sleep problems.

### Procedure

Participants completed the study online via Qualtrics. Upon providing informed consent, participants completed a screener question to ensure that they have played games in the past 12 months and the Background Information Form. Subsequently, participant completed the IGDS9-SF [32], the GDT [33], the Motives for Online Gaming Questionnaire [13], the Depression Anxiety Stress Scale [1], and the Single-Item Sleep Quality Scale [39]. These instruments were administered in a randomized order to control fatigue and order effects. Data collection was conducted and completed in August 2023. This procedure was conducted in accordance with the Declaration of Helsinki and was approved by the university’s Human Research Ethics Committee (Approval number: H9100).

## Results

The results were analyzed using SPSS Version 21 with the alpha level set at 0.05. For the time spent playing games on a weekday or single weekend day variables, 43 (2.1%) out of the 2014 values were more than 24 h, suggesting that participants might have misunderstood the question. These values were removed for subsequent analyses.

### Dropout Analyses

Dropout analyses were conducted to examine differences on demographic and gaming-related information between those with missing data on one or more instruments and the final sample. A series of *t*-tests showed that those with missing data (*M* = 26.54, *SD* = 6.18) were significantly younger than the final sample (*M* = 28.46, *SD* = 6.20), *t*(1153) = 3.51, *p* < .001. Furthermore, those with missing data (*M* = 3.34, *SD* = 1.68) spent less time playing games on weekends than the final sample (*M* = 4.31, *SD* = 3.22), *t*(38.74) = 3.09, *p* = .004. There were no significant differences for time spent playing games on weekdays and money spent on games in a month. A series of chi-square tests of independence showed that those with missing data were more likely to be male (60.1% vs. 49.5%), χ^2^(2, *n* = 1151) = 6.33, *p* = .042, to be Malay (27.3% vs. 13.4%), χ^2^(3, *n* = 1151) = 33.91, *p* = < 0.001, to live in a 3-room Housing and Development Board flat (38.5% vs. 27.3%), χ^2^(7, *n* = 1150) = 18.84, *p* = .009, and to have post-secondary (non-tertiary) education level (11.9% vs. 6.2%), χ^2^(4, *n* = 1132) = 12.92, *p* = .012, compared with the final sample. There were no significant associations for nationality, occupation, and favorite game genre.

### Missing Values Analysis

The remaining data for the instruments was subjected to Little’s [28] Missing Completely at Random test. The percentage of missing data across the variables was 0.1%. The test suggested that the data was missing completely at random, all *p* values > 0.05. Consequently, the expectation-maximization method was used to address the problem of missing data [14]. A total of seven values were imputed.

### IGD and GD

IGD prevalence was 10.3% (*n* = 104, 95% CI = 8.4, 12.2) and GD prevalence was 5.0% (*n* = 50, 95% CI = 3.6, 6.3). The descriptives of each criterion of IGD and GD are presented in Table 3. For IGD, the most often endorsed criterion was relieve negative moods (*n* = 355, 25.3%) and the least often endorsed criterion was withdrawal (*n* = 124, 12.3%). For GD, the most often endorsed criterion was continued gaming despite problems (*n* = 173, 17.2%) and the least often endorsed criterion was impaired control over gaming (*n* = 131, 13.0%).

**Table 3:** Descriptives of each Criterion of Internet Gaming Disorder (IGD) and Gaming Disorder (GD)

					Endorsement of Rating (%)					
No.	Criterion and Item		*M*	*SD*	Never	Rarely	Sometimes	Often	Very Often	Endorsement of Criterion (%)
	IGD									
1	Preoccupation		2.54	1.11	20.6	28.1	33.6	12.3	5.4	17.7
		Do you feel preoccupied with your gaming behaviour? (Some examples: Do you think about previous gaming activity or anticipate the next gaming session? Do you think gaming has become the dominant activity in your daily life? )								
2	Withdrawal		2.24	1.07	30.3	30.2	27.3	9.8	2.5	12.3
		Do you feel more irritability, anxiety or even sadness when you try to either reduce or stop your gaming activity?								
3	Tolerance		2.41	1.15	28.0	24.8	29.9	12.8	4.6	17.4
		Do you feel the need to spend increasing amount of time engaged gaming in order to achieve satisfaction or pleasure?								
4	Unsuccessful attempts to stop		2.31	1.13	30.1	28.4	26.1	11.5	4.0	15.5
		Do you systematically fail when trying to control or cease your gaming activity?								
5	Loss of interest in other activities		2.29	1.18	32.7	27.1	23.1	12.4	4.7	17.1
		Have you lost interests in previous hobbies and other entertainment activities as a result of your engagement with the game?								
6	Continued gaming despite problems		2.21	1.16	35.6	26.1	23.8	10.3	4.2	14.5
		Have you continued your gaming activity despite knowing it was causing problems between you and other people?								
7	Deception		2.00	1.17	48.2	20.1	18.0	10.6	3.1	13.7
		Have you deceived any of your family members, therapists or others because the amount of your gaming activity?								
8	Relieve negative moods		2.67	1.22	22.9	20.4	31.3	17.8	7.5	25.3
		Do you play in order to temporarily escape or relieve a negative mood (e.g., helplessness, guilt, anxiety)?								
9	Loss of a relationship or job		1.96	1.22	53.3	16.0	16.3	10.4	4.1	14.5
		Have you jeopardised or lost an important relationship, job or an educational or career opportunity because of your gaming activity?								
	GD									
1	Impaired control over gaming		2.25	1.10	30.5	30.5	26.1	9.1	3.9	13.0
		I have had difficulties controlling my gaming activity.								
2	Increasing priority given to gaming		2.31	1.10	28.9	28.1	29.2	10.5	3.4	13.9
		I have given increasing priority to gaming over other life interests and daily activities.								
3	Continued gaming despite problems		2.31	1.17	31.9	26.8	24.1	12.7	4.5	17.2
		I have continued gaming despite the occurrence of negative consequences.								
4	Impairment in various life domains		2.07	1.21	44.7	22.5	19.3	7.5	5.9	13.4
		I have experienced significant problems in life (e.g., personal, family, social, education, occupational) due to the severity of my gaming behavior.								

Note. IGD = Internet Gaming Disorder; GD = Gaming Disorder; Responses given as 4 or 5 (*Often* or *Very Often*) are coded as an endorsement of the criterion

Because of the disparity in prevalence rates in the two sets of diagnostic criteria, A Chi-square test of independence was conducted to examine their association. The Chi-square test of independence (with Yates Continuity Correction) found a significant association between IGD prevalence and GD prevalence, χ^2^(1, *n* = 1008) = 209.37, *p* < .001, *phi* = 0.46 (large effect). Despite the significant association, an inspection of the cross tabulation (see Table 4) showed that 82 participants (8.1%) were miscategorized: 68 (6.7%) met the IGD criteria but not the GD criteria whereas 14 (1.4%) met the GD criteria but not the IGD criteria. Only 36 participants (3.6%) met both the IGD and GD criteria.

**Table 4:** Association between Internet Gaming disorder (IGD) prevalence and gaming disorder (GD) prevalence, n (%)

	GD Prevalence		
IGD Prevalence	Yes	No	Total
Yes	36 (3.6%)	68 (6.7%)	104 (10.3%)
No	14 (1.4%)	890 (88.3%)	904 (89.7%)
Total	50 (5.0%)	958 (95%)	1008 (100%)

### Correlates to IGD and GD

A total of seven participants (0.7%) preferred not to indicate their gender and were excluded from analyses involving the variable. A series of two Chi-square test of independence (with Yates Continuity Correction) found significant associations between gender, and IGD prevalence, χ^2^(1, *n* = 1001) = 18.31, *p* < .001, *phi* = − 0.14 (small effect), and GD prevalence, χ^2^(1, *n* = 1001) = 9.42, *p* < .01, *phi* = − 0.10 (small effect). Specifically, the IGD prevalence was 14.6% (*n* = 73, 95% CI = 11.5, 17.7) for males and 6.2% (*n* = 31, 95% CI = 4.1, 8.3) for females. In addition, the GD prevalence was 7.2% (*n* = 36, 95% CI = 4.9, 9.5) for males and 2.8% (*n* = 14, 95% CI = 1.3, 4.2) for females.

A series of *t*-tests were conducted to examine the effects of IGD prevalence and GD prevalence on gaming time, gaming motivations, negative emotional states, and sleep quality. The results are presented in Tables 5 and 6, respectively. Participants with IGD spent more time playing games on weekdays and weekends, had higher scores on the seven gaming motivations (social, escape, competition, coping, skill development, fantasy, and recreation) and three negative emotional states (depression, anxiety, and stress) than their counterparts without IGD, all *p* values < 0.001. The effects of IGD prevalence on sleep quality were close to statistical significance. Participants with IGD had higher sleep quality than their counterparts without IGD, *p* = .05. Similar results were found for GD prevalence. Participants with GD spent more time playing games on weekdays and weekends, had higher scores on the seven gaming motivations, three negative emotional states, and sleep quality than their counterparts without GD, all *p* values < 0.001 except for sleep quality where *p* = .029.

**Table 5:** Mean (Standard Deviation) of Gaming Time, Gaming motivations, negative Emotional States, and Sleep Quality for participants with internet gaming disorder (IGD)

		IGD Prevalence			Group Differences	
Variables		Yes	No		*t*(*df*)	*p*
Gaming Time						
	Weekday	4.52 (3.91)	2.82 (2.53)		−4.22 (107.49)	< 0.001
	Weekend	6.46 (4.83)	4.07 (2.89)		−4.85 (107.07)	< 0.001
Gaming Motivations						
	Social	15.24 (3.53)	9.97 (3.68)		−13.89 (1006)	< 0.001
	Escape	15.84 (2.67)	10.95 (4.04)		−16.58 (162.81)	< 0.001
	Competition	15.38 (3.33)	10.58 (3.90)		−13.65 (137.77)	< 0.001
	Coping	15.72 (2.83)	11.21 (3.72)		−14.86 (147.45)	< 0.001
	Skill Development	15.73 (3.16)	10.95 (3.92)		−14.22 (142.21)	< 0.001
	Fantasy	15.73 (3.01)	10.38 (4.13)		−16.43 (151.50)	< 0.001
	Recreation	12.02 (2.32)	9.67 (3.09)		−9.41 (148.77)	< 0.001
Negative Emotional States						
	Depression	12.39 (2.90)	7.48 (3.06)		−16.27 (130.87)	< 0.001
	Anxiety	11.98 (2.70)	7.28 (2.72)		−16.69 (1006)	< 0.001
	Stress	12.24 (2.46)	7.71 (2.83)		−17.51 (136.57)	< 0.001
Sleep Quality		7.28 (2.35)	6.81 (1.73)		−1.98 (116.26)	= 0.05

**Table 6:** Mean (Standard Deviation) of Gaming Time, Gaming motivations, negative Emotional States, and Sleep Quality for participants with Gaming Disorder (GD)

		GD Prevalence			Group Differences	
Variables		Yes	No		*t*(*df*)	*p*
Gaming Time						
	Weekday	5.43 (4.70)	2.88 (2.57)		−3.62 (45.28)	< 0.001
	Weekend	6.70 (4.38)	4.19 (3.11)		−3.88 (48.31)	< 0.001
Gaming Motivations						
	Social	16.66 (3.30)	10.20 (3.76)		−11.91 (1006)	< 0.001
	Escape	16.50 (2.50)	11.19 (4.09)		−14.05 (63.59)	< 0.001
	Competition	16.04 (4.05)	10.82 (3.95)		−9.10 (1006)	< 0.001
	Coping	16.50 (2.67)	11.41 (3.77)		−13.06 (59.69)	< 0.001
	Skill Development	16.88 (3.25)	11.16 (3.96)		−12.00 (56.87)	< 0.001
	Fantasy	16.98 (2.96)	10.61 (4.17)		−14.48 (59.65)	< 0.001
	Recreation	12.50 (2.04)	9.78 (3.09)		−8.90 (61.33)	< 0.001
Negative Emotional States						
	Depression	13.30 (2.71)	7.71 (3.19)		−14.06 (56.30)	< 0.001
	Anxiety	12.58 (2.91)	7.52 (2.87)		−12.17 (1006)	< 0.001
	Stress	12.82 (2.60)	7.94 (2.95)		−11.48 (1006)	< 0.001
Sleep Quality		7.64 (2.55)	6.82 (1.76)		−2.25 (51.45)	= 0.029

## Discussion

The current study provided a snapshot of the state of gaming in Singapore in 2023. First, the results showed a prevalence rate of 10.3% for IGD and 5.0% for GD. The IGD prevalence rate was higher than meta-analytic studies that found pooled prevalence rates of 5.08% [41] and 6.30% [22] for participants from Asian regions. However, it was comparable to the pooled prevalence rate of 10.40% for young adult participants [17]. In contrast, it was lower than the prevalence rate of 17.70% for participants from Singapore [43]. This might be due to the study’s inclusion of adolescents, an at-risk population, resulting in a higher prevalence rate. The GD prevalence rate was higher than a previous study that found a prevalence rate of 1.80% only [33]. Overall, the relatively high prevalence rate of IGD and GD in the current study indicates that problematic gaming is a social issue among young adults in Singapore that deserves greater attention from researchers, practitioners, and the government.

The prevalence rate of 10.3% for IGD was twice as high as the prevalence rate of 5.0% for GD in the current study. Furthermore, despite a significant association between the two prevalence rates, a total of 82 participants (8.1%) were miscategorized. This might be explained by the differences in diagnostic criteria between the DSM-V IGD criteria [2] and ICD-11 GD criteria [50]. Specifically, the DSM-V IGD criteria might be overly sensitive, resulting in healthy gamers meeting its diagnostic criteria. Alternatively, the ICD-11 GD criteria might not be sufficiently sensitive, resulting in problematic gamers not meeting its diagnostic criteria. Either way, given the ethical implications and ramifications of a misdiagnosis, it is clear that more research is needed to explain the disparity in prevalence rates.

Second, the results showed that males are twice as likely to engage in problematic gaming than females. Specifically, males had a IGD prevalence rate of 14.6% and a GD prevalence rate of 7.2%. In contrast, females had a IGD prevalence rate of 6.2% and a GD prevalence rate of 2.8%. This was consistent with previous studies that found males to be at higher risk for IGD than females [11, 31, 41] and females are more predisposed towards excessive social media usage [5]. The higher gaming prevalence observed in males might be explained by the presence of (a) competitive elements (e.g., leaderboards to rank the top 10 gamers), (b) opportunities for socially acceptable expressions of aggression (e.g., game modes where a gamer can ‘kill’ other gamers), and (c) sexualized content (e.g., hypersexualized female avatars) in games [29, 42]. Consequently, males might be more attracted to games than females, resulting in a higher risk for problematic gaming.

Third, participants with IGD or GD reported higher weekday and weekend gaming time than their counterparts with no IGD or GD. This was consistent with a previous study that found a positive association between gaming time and the number of endorsed DSM-V IGD criteria [33]. This might be explained by a longer gaming time serving as both a risk factor and negative consequence of problematic gaming. For example, playing games for an extended period of time precludes spending time on important life domains (e.g., family, work, etc.), increasing the risk of impairment in those domains. Furthermore, individuals with problematic gaming might be playing games longer due to the combined effects of both withdrawal and tolerance. Taken together, gaming time is closely related to problematic gaming and could be the target for interventions [6] for an abstinence program for problematic gaming).

Fourth, participants with IGD or GD also reported higher scores on the seven gaming motivations (social, escape, competition, coping, skill development, fantasy, and recreation) than their counterparts with no IGD or GD. This was consistent with previous studies that found positive correlations between the factors of gaming motivations and IGD [27, 35]. This might be due to the close links between some of the motivations and problematic gaming. For example, the escape, coping, and fantasy motivations appeared to be related to the DSM-V IGD criterion of gaming to escape or to relieve negative moods [2]. In particular, the escape motivation is relatively important since existential concerns (e.g., concerns about death, isolation, meaningless, etc.) might underline problematic gaming [37]. Indeed, participants reminded of their mortality spent almost twice as long on a game than control participants [10]. As evidence accumulates for the role of existential concerns in problematic gaming, clinicians could consider the use of existential therapy as an intervention for problematic gaming. Finally, the social, competition, and skill development motivations might result in longer gaming times, which increases the risk for problematic gaming. Overall, an understanding of why people play games could provide us with insights into problematic gaming.

Fifth, the results showed that participants with IGD or GD reported higher scores on the three negative emotional states (depression, anxiety, and stress) than their counterparts with no IGD or GD. This was consistent with previous studies that found positive correlations between negative emotional states and IGD [4, 44, 49]. Negative emotional states and problematic gaming could affect each other in a bidirectional manner. For example, individuals experiencing negative emotional states might play games as a form of escape. However, playing games for an extended period of time could lead to impairment in important life domains. In turn, the impairment might lead to negative emotional states. This vicious cycle could play a critical role in maintaining or exacerbating symptoms of problematic gaming.

Finally, participants with IGD or GD also reported higher scores on sleep quality than their counterparts with no IGD or GD. This was inconsistent with previous studies that found negative correlations between sleep quality and IGD [25, 49]. This might be due to a genuine experience of higher sleep quality. For example, individuals might engage in problematic gaming to relieve negative moods, resulting in a higher sleep quality. Alternatively, the results might be due to the use of a single item to assess sleep quality. The Single-Item Sleep Quality Scale was used due to its brevity and good psychometric properties [39]. However, the single item might not be sufficient to capture the different components of sleep quality like sleep latency, sleep duration, sleep efficiency, and sleep disturbances [7]. Future research could examine if problematic gaming is differentially associated with the different components of sleep quality.

Limitations of the study should be noted. First, there are numerous arguments against the inclusion of problematic gaming in the DSM-V and ICD-11 [15, 46]. Furthermore, even among researchers who accept the validity of problematic gaming as a disorder, they disagree on the validity of some of the diagnostic criteria [9, 18]. Since the current study used the DSM-V and ICD-11 criteria, it is expected that the results will change if there are any modifications to the criteria. Second, the current study used a cross-sectional correlational design and was unable to establish cause-and-effect relationships. For example, while it was assumed that gaming motivations serve as risk factors for problematic gaming, it is possible that they could be outcomes of problematic gaming too. In the future this limitation might be controlled by using a longitudinal or experimental design.

In conclusion, although more research is needed to explain the disparity in prevalence rates for IGD and GD, the relatively high prevalence rates in the current study suggested that problematic gaming is a social issue in Singapore that deserves greater attention. The high rates also suggest that there is a need for more interventions to regulate excessive gaming behaviors. Furthermore, with the exception of sleep quality, known risk factors and negative consequences of problematic gaming were also found in the current study. Future research could develop and evaluate prevention programs based on known risk factors, or intervention programs to alleviate the symptoms and negative consequences of problematic gaming.

## Electronic Supplementary Material

Below is the link to the electronic supplementary material.

Supplementary Material 1

## Data Availability

The data associated with the manuscript can be found on the Open Science Framework: https://osf.io/d9fct/?view_only=cb01055a51b14a9bafbd5413bef31910.

## Footnotes

1 Given the numerous terminologies in the literature, the current paper uses “problematic gaming” to refer to the condition generally, “IGD” to refer to the condition as assessed by the DSM-V, and “GD” to refer to the condition as assessed by the ICD-11.
