# Supplementary material for: Prevalence and Correlates of (Internet) Gaming Disorder among Young Adults in Singapore
Source: Psychiatr Q. 2025 Feb 12;96(2):345–63. doi: 10.1007/s11126-025-10119-9 (PMC12213995; doi:10.1007/s11126-025-10119-9)
Supplement: Supplementary file 1 — Supplementary Material 1 [file 11126_2025_10119_MOESM1_ESM.docx]

**Declaration of Interests**

The authors declare that they have no known competing financial interests or personal relationships that could have appeared to influence the work reported in this paper.
